# Aldo-Keto Reductases 1B in Adrenal Cortex Physiology

**DOI:** 10.3389/fendo.2016.00097

**Published:** 2016-07-22

**Authors:** Emilie Pastel, Jean-Christophe Pointud, Antoine Martinez, A. Marie Lefrançois-Martinez

**Affiliations:** ^1^Diabetes and Obesity Research Group, University of Exeter Medical School, Exeter, UK; ^2^CNRS, UMR 6293/INSERM U1103, Génétique, Reproduction et Développement, Clermont Université, Aubière, France

**Keywords:** aldose reductase, adrenal physiology, AKR1B

## Abstract

Aldose reductase (AKR1B) proteins are monomeric enzymes, belonging to the aldo-keto reductase (AKR) superfamily. They perform oxidoreduction of carbonyl groups from a wide variety of substrates, such as aliphatic and aromatic aldehydes or ketones. Due to the involvement of human aldose reductases in pathologies, such as diabetic complications and cancer, AKR1B subgroup enzymatic properties have been extensively characterized. However, the issue of AKR1B function in non-pathologic conditions remains poorly resolved. Adrenal activities generated large amount of harmful aldehydes from lipid peroxidation and steroidogenesis, including 4-hydroxynonenal (4-HNE) and isocaproaldehyde (4-methylpentanal), which can both be reduced by AKR1B proteins. More recently, some AKR1B isoforms have been shown to be endowed with prostaglandin F synthase (PGFS) activity, suggesting that, in addition to possible scavenger function, they could instigate paracrine signals. Interestingly, the adrenal gland is one of the major sites for human and murine AKR1B expression, suggesting that their detoxifying/signaling activity could be specifically required for the correct handling of adrenal function. Moreover, chronic effects of ACTH result in a coordinated regulation of genes encoding the steroidogenic enzymes and some AKR1B isoforms. This review presents the molecular mechanisms accounting for the adrenal-specific expression of some AKR1B genes. Using data from recent mouse genetic models, we will try to connect their enzymatic properties and regulation with adrenal functions.

## Introduction

The pituitary adrenocorticotropic hormone is the main regulator of adrenal steroidogenesis acting through the cAMP-dependent protein kinase (PKA) signaling pathway. The fixation of ACTH second messenger, cAMP, on PKA regulatory subunits, leads to the release of catalytic subunits that in turn phosphorylate several targets, including transcription factors, such as the CCAAT enhancer-binding protein (C/EBP) and the cAMP response element-binding protein (CREB). Acting coordinately with tissue-specific factors such as the steroidogenic factor 1 (SF1), they stimulate expression of genes encoding enzymes and proteins involved in cholesterol metabolism, mobilization, and transport. In the adrenal cortex, steroidogenesis activation results in the generation of large amount of lipid aldehydes i.e., isocaproaldehyde (4-methylpentanal) produced by the CYP11A1 cholesterol side-chain cleavage and 4-hydroxynonenal (4-HNE), whose harmfulness has to be supported by coordinately regulated detoxifying enzymes. We and other groups have previously observed that the adrenal gland is one of the main sites of expression of both murine and human AKR1B proteins (1–3). Aldose reductases are cytosolic monomeric enzymes, belonging to the aldo-keto reductase (AKR) superfamily. This superfamily encompasses more than 150 NAD(P)(H)-dependent oxidoreductases distributed in all prokaryotic and eukaryotic kingdoms, including yeast, plant, invertebrates, and vertebrates. They catalyze the reduction of carbonyl groups from a wide variety of substrates, such as aliphatic and aromatic aldehydes, ketones, keto prostaglandins, ketosteroids, and xenobiotics. Based on sequence identity, these proteins are divided in 15 families termed AKR1–AKR15, each family having less than 40% amino acid sequence identity with the others (4–6).

Among the AKR1 family, the aldose reductase subgroup-designated AKR family 1 member B (AKR1B) is one of the most characterized because of its involvement in human diseases, such as diabetic complications resulting from the ability of the former AKR family 1 member B1 (AKR1B1) to reduce glucose into sorbitol in a NADPH + H^+^-dependent manner during hyperglycemia. In addition to glucose conversion, AKR1B proteins display multiple other activities, including reduction of aldehyde group of by-products derived from lipid peroxidation or steroid synthesis, retinoids, xenobiotics, and prostaglandins (1, 7–9). The AKR1B subfamily includes proteins sharing a high degree of similarity (i.e., more than 65% of identity; Table 1). They are organized in two subgroups based on their ability to reduce glucose: aldose reductases (AR; AKR1B1–6) and aldose reductase-like proteins (ARLP; Akr1b7–19), respectively (4, 10–12). Their structure, enzymatic properties, and substrate specificities have been the subject of many studies (1, 5, 7, 13–17), emphasizing that in addition to their high percentage of identity, they also display redundant substrate specificities and overlapping expression patterns. These potential redundancies, then, complicate study of their distinct biological functions in specific physiological or pathological processes. Analysis of murine genetic models and identification of the mechanisms regulating their expression are the necessary steps to complete our understanding in AKR1Bs biological function.

**Table 1:** **Comparison of protein sequence identity (%) between human (h) and murine (m) AKR1B proteins**.

		Aldose reductases		Aldose reductase-like proteins				
	AKR1	B1	b3	b7	b8	B10	B15	b16
Aldose reductases	B1 (h)	100	85.8	71.6	70.7	71	65.1	70.7
	b3 (m)	85.8	100	69.7	69.4	70.7	65.4	70.3

Aldose reductase-like proteins	b7 (m)	71.8	69.7	100	82.3	79.8	72.1	84.8
	b8 (m)	70.7	69.4	82.3	100	82.3	74.9	82.6
	B10 (h)	65.1	70.7	79.8	82.3	100	86	82.9
	B15 (h)	65.1	65.4	72.1	74.9	86	100	76.2
	b16 (m)	70.7	70.3	84.8	82.6	82.9	76.2	100

*The protein sequences were aligned using the Clustal Omega program. The amino acid sequences used to achieve this multiple alignment correspond to the accession numbers listed in Table 2*.

*The Gray shade highlights the necessary 100% identity between 2 identical protein sequences*.

This review will provide an updated integrative view on specific regulations of human and murine aldose reductase genes with enzymatic and functional data in the adrenal gland physiology [further information on AKR1Bs in other endocrine functions is reviewed in Ref. (18)]. Since several studies allowed identification of some murine and human aldose reductase genes as orthologs, common features will be presented for each corresponding pair, and individual isoform specificities will be discussed.

## Human and Murine AKR1B Gene Synopsis

### Human AKR1B Genes

**Figure 1:**
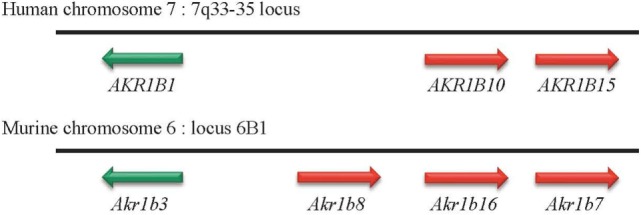
**Genomic organization of AKR1B genes in humans and mice**. In human and mouse genomes, genes encoding aldose reductase are located on chromosomes 7 and 6, respectively. Whatever the case, these genes are organized in tandem.

Three human AKR1B genes organized in tandem on chromosome 7q33–35 have been identified (Table 2; Figure 1): *AKR1B1* [human aldose reductase (19)], *AKR1B10* [also designated as HSI reductase: human small intestine reductase (1, 7)], and *AKR1B15* (20). *AKR1B1* seems to be ubiquitously expressed, whereas *AKR1B10* expression was only reported in small intestine, colon, liver, thymus, and adrenal gland (1, 7). *AKR1B15* gene was recently characterized and identified as closely related to the *AKR1B1* and *AKR1B10* cluster on chromosome 7 (Figure 1). *AKR1B15* undergoes alternative splicing, giving rise to two protein isoforms, designated as AK1R1B15.1 and AKR1B15.2, expressed in thyroid gland and testis, respectively, and both in adipose tissue and placenta. *AKR1B15.1* transcript encodes a putative protein sharing 68 and 91% sequence identity with AKR1B1 and AKR1B10, respectively (21). Both *AKR1B15* transcripts were absent from human adrenal (20).

**Table 2:** **Human and murine members of the aldo-keto reductase B1 subgroup (AKR1B)**.

Symbol	Common associated protein designation	Species	ARN	Protein
AKR1B1	Aldose reductase	Human	NM_001628	NP_001619
Ark1b3	Aldose reductase	Mouse	NM_009658	NP_033788
Akr1b7	Mouse *vas deferens* protein (MVDP)	Mouse	NM_009731	NP_033861
Akr1b8	Fibroblast growth factor-regulated protein 1 (FR-1)	Mouse	NM_008012	NP_032038
AKR1B10	Small intestine reductase (HSI)	Human	NM_020299	NP_064695
AKR1B15	Aldose reductase (putative)	Human	NM_001080538	NP_001074007
Akr1b16	Aldose reductase (putative)	Mouse	NM_172398	NP_765986

### Murine Akr1b Genes

Four murine Akr1b genes have been described: *Akr1b3* (murine aldose reductase), *Akr1b7* [previously named MVDP: mouse vas deferens protein (22)], *Akr1b8* [previously named FR-1: fibroblast growth factor (FGF)-related protein (23)], and *Akr1b16* (21) (Table 2). Murine aldose reductase genes are located on chromosome 6 (locus 6B1), and their tandem arrangement suggests (as for the three human *AKR1Bs*) that these four genes arise from an ancestral gene duplication event (10, 12) (Figure 1). Several studies had allowed identification of murine *Akr1b3, Akr1b8* as the orthologs of the human *AKR1B1* and *AKR1B10*, respectively. However, this phylogenetic analysis between human and mouse AR has some limits and will be commented below. *AKR1B1, Akr1b3*, and *Akr1b16* are rather ubiquitously expressed (11, 21), whereas *Akr1b7* and *Akr1b8* exhibit a restricted tissue distribution. Indeed, *Akr1b7* is detected in vas deferens, adrenal glands, gonads, intestine, white adipose tissue, eye, liver, and kidney (2, 22, 24–26) and *Akr1b8* in testis, heart, adrenal glands, intestine, and liver (2, 11, 23).

## AKR1B in Adrenals: Between Detoxification and Paracrine Signaling

### Akr1b3/AKR1B1: Expression Pattern and Relevant Functions

In studies using murine adrenal cell lines (Y1 adrenocortical cells and MPC862L chromaffin cells), we found that Akr1b3 protein accumulates in both adrenal cortex and medulla. Moreover, *in vivo* and *ex vivo* hormonal manipulations demonstrated that unlike the other murine Akr1b7 and Akr1b8 isoforms, Akr1b3 is expressed in the whole gland (27). Finally, cAMP stimulation failed to modulate *Akr1b3* expression in Y1 cell line, confirming that *Akr1b3* was insensitive to ACTH signaling (28) (Table 3).

**Table 3:** **Localization and regulation of AKR1B in adrenal gland**.

Isoforms	Localization	Analyses	Control by ACTH/cAMP	Transcriptional regulators	Reference
**Human**					
AKR1B1	Cortex	IHC, RNA master blot	+	n.d.	(1, 27)
AKR1B10	Adrenal^a^	RNA master blot	n.d.	n.d.	(1)
AKR1B15	n.d.	n.d.	n.d.	n.d.	–
**Mouse**					
Akr1b3	Cortex and medulla	WB	No	No	(27, 28)
Akr1b7	Cortex	NB, WB, IHC, ISH	+	Sp1, C/EBPβ, SF1	(2, 27, 28, 55, 58, 67)
Akr1b8	Cortex	WB, ISH	No	No	(2, 27, 28)
Akr1b16	n.d.	n.d.	n.d.	n.d.	–

*^a^Intra-adrenal tissue localization was not specified*.

*n.d., not determined; NB, Northern Blot; WB, Western Blot; RT–PCR, reverse transcription–polymerase chain reaction; ISH, in situ hybridization; IHC, immunohistochemistry; n.d., not determined*.

Considering their enzymatic properties and expression levels in murine adrenal cortex, Akr1b7 and Akr1b8 are considered as the main isocaproaldehyde reductase and 4-HNE reductase, respectively, while Akr1b3 could rather participate in the elimination of these toxic compounds in basal physiological conditions (27–29). Moreover, Akr1b3 also displays 9-,11-endoperoxide reductase activity that, when coupled to COX-1 (cyclooxygenase type 1), allows prostaglandin F2α (PGF_2α_) synthesis in adrenal cortex and medulla (see below).

Despite all these evidences upon Akr1b3 involvement in both lipid aldehyde detoxification and PGF_2α_ synthesis, *in vivo Akr1b3* gene invalidation (*Akr1b3*^−^*^/^*^−^ mice) did not highlight any phenotype related to adrenal gland (30, 31). The lack of adrenal dysfunction in *Akr1b3*^−^*^/^*^−^ mice may result from the redundancy of enzymatic properties carried by the other murine isoforms expressed in the gland (Akr1b7 and Akr1b8) that could then compensate Akr1b3 loss.

**Figure 2:**
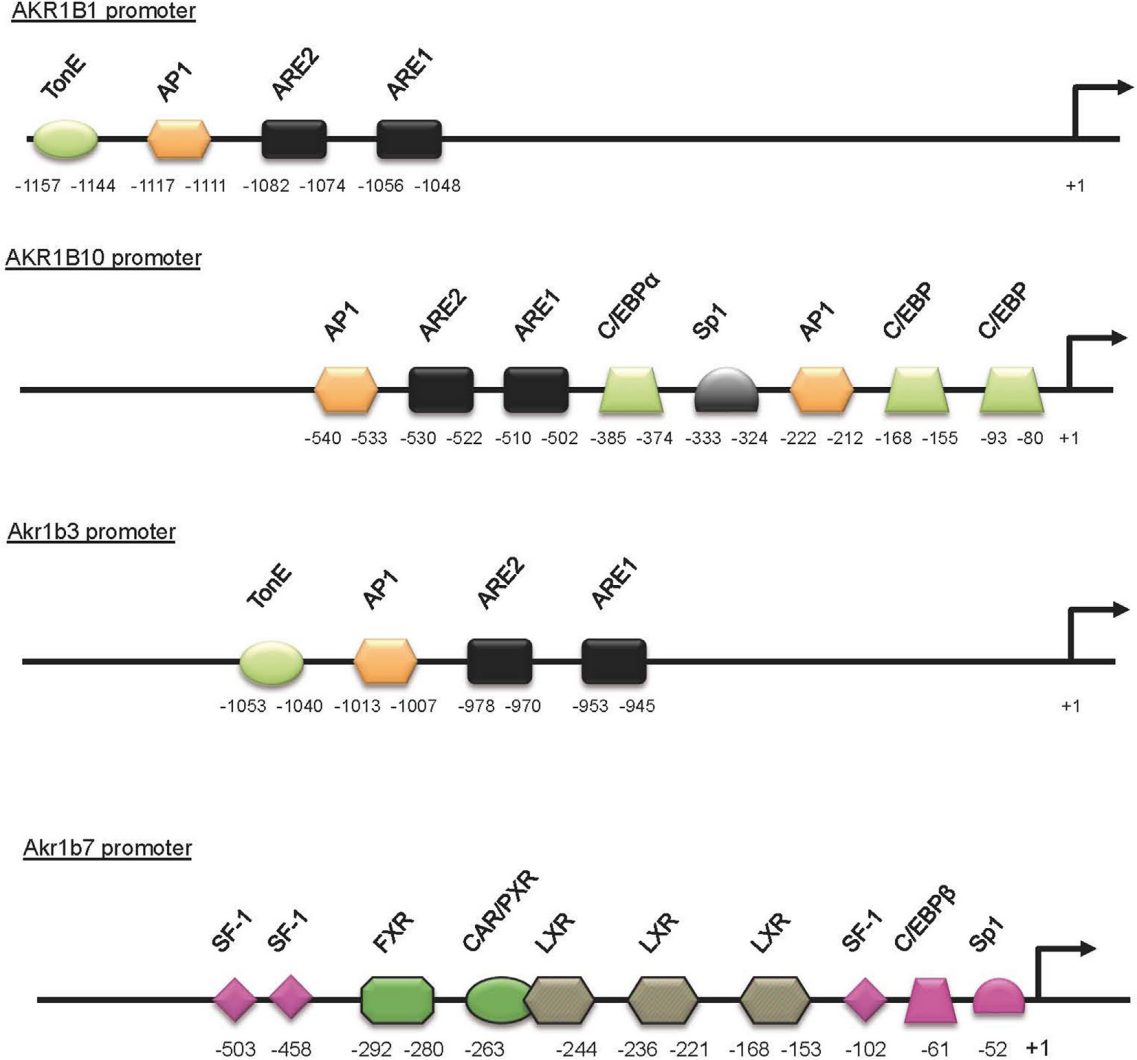
**Schematic representation of the *AKR1B1, AKR1B10, Akr1b3*, and *Akr1b7* promoters**. *Cis*- and *trans*-acting factors shown to be involved in stress responsiveness are indicated. The DNA-binding site for transcription factors and nuclear receptors required for the *Akr1b7*-specific expression in adrenal gland (pink boxes) are shown. LXR-binding sites are involved in both adrenal and intestine *Akr1b7* expression. TonE, tonicity response element; AP1, activator protein 1 binding site; ARE, antioxidant response element; C/EBP, CCAAT enhancer binding protein binding site; Sp1, selective promoter factor 1 binding site.

In human adrenal gland, *AKR1B1* transcripts have been initially detected using RNA master blot (1) (Table 3). Thereafter, using immunohistochemistry, we confirmed those results and demonstrated that AKR1B1 expression pattern is restricted to the cortex of adrenal gland (27). Treatment of the human adrenocortical tumor cells NC1-H295 with forskolin (adenylyl cyclase inducer) allowed us to suggest that similar to the murine isoform Akr1b7, *AKR1B1* expression was sensitive to ACTH (32) (Table 3). The molecular mechanisms and *cis*-acting elements responsible for ACTH/cAMP responsiveness of *AKR1B1* gene have not been investigated to date (Figure 2). Analysis of *AKR1B1* expression in stress-related disorders was not explored to date. Moreover, analysis of adrenal samples from Cushing’s disease (ACTH-producing pituitary tumor) revealed unchanged mRNA levels of *AKR1B1* gene (32).

**Figure 3:**
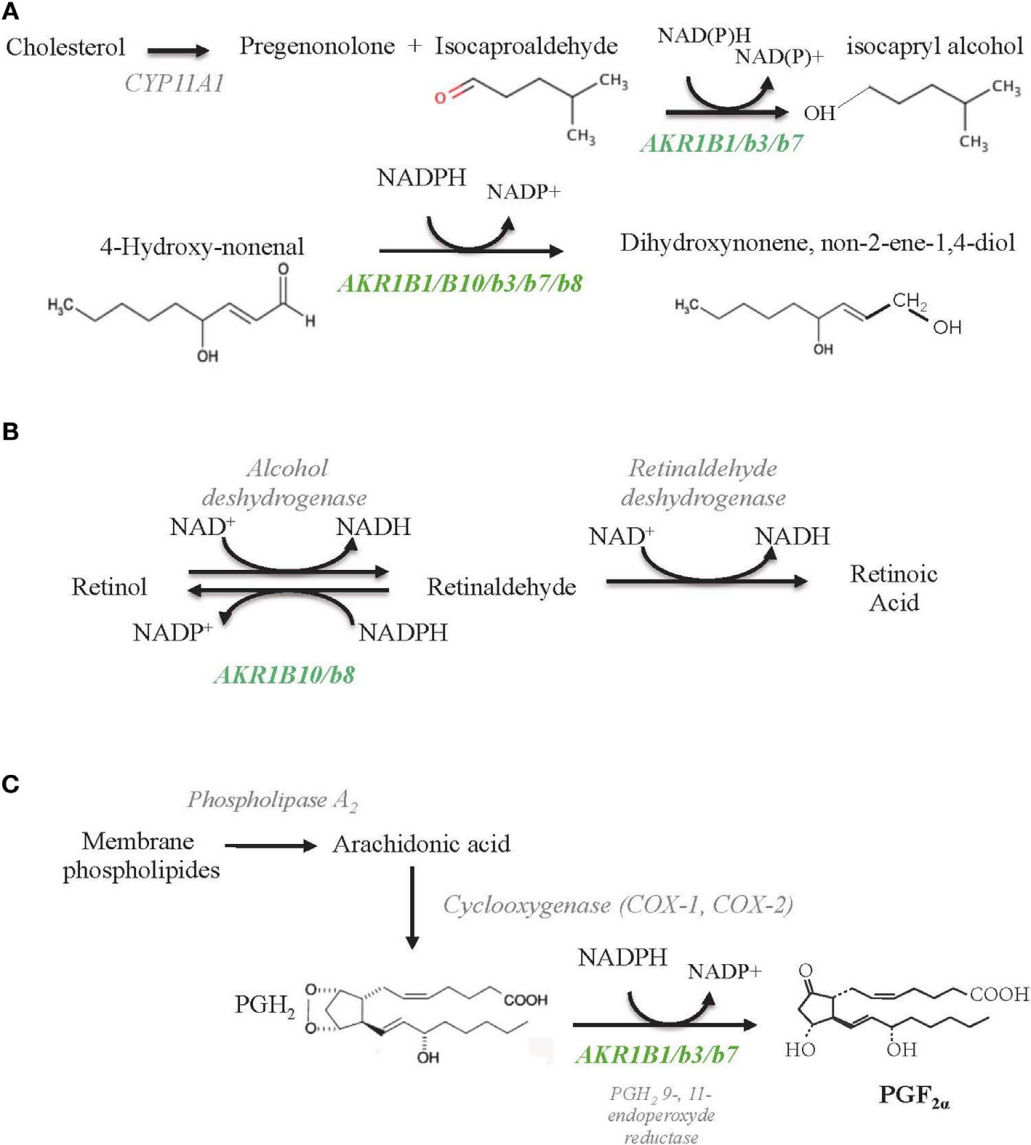
**Schematic diagrams of enzymatic reactions illustrating AKR1B involvement toward lipid aldehyde detoxification (A), retinoids (B), and prostaglandins (C) metabolism**. All the indicated substrates and enzymatic activities are supposed to coexist in the adrenal glands, but their relative importance in adrenal function remains unknown so far. AKR1B isoforms endowed with each of these activities are indicated in green.

Based on its enzymatic properties, AKR1B1 has long been considered as the sole isocaproaldehyde reductase in the human adrenal gland (33) (Figure 3A; Table 4). Interestingly, unlike murine Akr1b7 isoform, NADPH-dependent isocaproaldehyde reductase activity carried by AKR1B1 was inhibited by tolrestat, a potent and specific aldose reductase inhibitor belonging to the carboxylic acids group of AR inhibitors (13, 29, 33). We demonstrated that AKR1B1 was also able to convert PGH_2_ into PGF_2α_ (34) (Figure 3C; Table 4). This 9-,11-endoperoxide reductase activity is also strictly NADPH-dependent and inhibited by tolrestat.

**Table 4:** **Kinetic constants of AKR1B toward isocaproaldehyde, 4-hydroxynonenal, retinaldehyde, and prostaglandin H_2_**.

Substrates	Isocaproaldehyde		4-hydroxynonenal		All-*trans*-retinaldehyde		Prostaglandin H_2_	
	*K*_m_ (μM)	*k*_cat_ (s^−1^)	*K*_m_ (μM)	*k*_cat_ (s^−1^)	*K*_m_ (mM)	*k*_cat_ (min^−1^)	*K*_m_ (μM)	*V*_max_ (nmol/min/mg)
**Human**								
AKR1B1	1^a^	0.66^a^	716^d^	0.84^d^	1.1^e^	0.35^e^	1.9^f^	44^f^
AKR1B10	330^b^	0.72^b^	31^d^	2.01^d^	0.6^e^	27^e^	No activity^f^	
AKRB15	n.d.		2.2^g^	0.08^g^	1^g^	5.4^g^	n.d.	
**Mouse**								
Akr1b3	62^c^	1.3^c^	665^g^	0.82^d^	1.0^e^	0.52^e^	26^f^	26^f^
Akr1b7	320^c^	0.38^c^	256^g^	0.1^d^	0.5^e^	0.02^e^	53.4^f^	53.4^f^
Akr1b8	71^c^	0.03^c^	230^g^	3.18^d^	2.1^e^	0.05^e^	No activity^f^	
Akr1b16	n.d.		n.d.		n.d.		n.d.	

*n.d., not determined*.

*Reference: ^a^(33); ^b^(40); ^c^(28); ^d^(11); ^e^(12); ^f^(34); ^g^(14)*.

We observed that in the human adrenal gland, AKR1B1 and the cAMP-inducible COX-2 isoform were co-localized in steroidogenic cortical cells (27) (Table 3). Then, we can consider that human adrenal cortex has the potential to produce PGF_2α_ in response to ACTH surge. The shared properties of human AKR1B1 and mouse Akr1b7, such as hormonal regulation and reductase activity toward common substrates, prompted us to postulate that they can be considered as functional orthologs at least in the adrenal cortex (32). Moreover, increased levels of *AKR1B1* transcripts in human were observed in adrenocortical adenomas harboring glucocorticoid autonomous hypersecretion (32). The possibility that AKR1B1 prostaglandin F synthase (PGFS) activity could participate in an intra-adrenal feedback loop between endocrine activities of cortex and medulla in human adrenal gland remains to be explored.

Given the high expression of *AKR1B1* in the adrenal cortex, we evaluated alterations in its expression in association with human adrenal disorders. The relative abundance of *AKR1B1* mRNA was decreased in adrenocortical carcinomas (ACC) when compared to benign tumors, Cushing’s hyperplasia, or normal adrenals (32). These data were reinforced by de Reyniès et al., who demonstrated that decreased *AKR1B1* expression was associated with malignancy using an unsupervised clustering analysis of the human adrenal tumors transcriptome (35). This identified AKR1B1 as a potential negative marker for adrenocortical malignancy.

### Akr1b8/AKR1B10: Phylogeny, Regulation, and Enzymatic Specificities

*Akr1b8* mRNA was initially detected in both fetal and adult murine adrenal cortex but remained undetected in the medulla by *in situ* hybridization (2). In fibroblasts as well as in adrenocortical Y1 cell line, *Akr1b8* was previously shown to be controlled by the FGF and phorbol myristate acetate (PMA) (23, 28). On the contrary, dexamethasone-induced ACTH suppression did not influence *Akr1b8* mRNA and protein accumulation (27), suggesting that its biological function did not seem to be related to the ACTH-dependent steroidogenic activity present in adrenocortical cells (Table 3). Interestingly, in digestive tract organs, such as liver and small intestine, *Akr1b8* gene was recently showed to be a target of NF-E2-related factor2 (Nrf2), which mediates transcriptional response to oxidative stress by binding to antioxidant response element (ARE) sites (36). As expected, *Akr1b8* expression was downregulated in digestive tract from Nrf2 knockout mice. However, no adrenal phenotype was described in these mice, and neither oxidative stress nor Nrf2 have been involved in adrenal *Akr1b8* expression so far.

Previous studies demonstrated that some AKR1B proteins showed efficient catalytic activity for the reduction of the abundant and highly reactive lipid-derived aldehyde 4-hydroxy-2-nonenal and the phospholipid aldehydes (17). In particular, Akr1b8 displays the most efficient phospholipid aldehyde and HNE-reductase activity in mouse tissues (17, 28, 37) (Figure 3; Table 4). All these enzymatic data suggest that in the adrenal cortex, Akr1b8 isoform could be devoted to detoxify aldehyde lipids abundantly accumulated in this tissue (38). Furthermore, expression of an antisense RNA directed against *Akr1b7* suppressed isocaproaldehyde reductase activity in adrenocortical Y1 cells without any alteration of Akr1b8 protein accumulation (29). In view of its enzymatic features and constitutive expression, Akr1b8 is unlikely to be the principal isocaproaldehyde reductase in the adrenocortical gland (28). *Akr1b8* gene disruption in mice led to reduced lipid synthesis and diminished proliferation of colonic epithelial cells but had no evident effect on general appearance, body weight, and reproduction. However, *in vivo* adrenal Akr1b8 physiological role remains to be examined since *Akr1b8* gene disruption first report did not notice evident effect on the adrenal physiology (39).

Whether *Akr1b8* and human *AKR1B10* gene can be considered as ortholog is still a matter of debate since they share high sequence identity, and proteins display several close structural and enzymatic properties (11) (Tables 1 and 4). In contrast to *Akr1b8, AKR1B10* gene expression is not controlled by FGF (12). Although *AKR1B10* mRNA was initially detected in adrenal glands using a human RNA Master Blot, to date, there is no more information available on its *in situ* localization and transcriptional control in this organ (1). Moreover, *AKR1B10* gene expression pattern only partially overlaps that of *Akr1b8*, since *AKR1B10* transcripts are absent from heart, lung, or testis (7, 11).

Comparative studies demonstrated that AKR1B10 exhibits higher 4-HNE-reductase activity than AKR1B1, while lower than the murine Akr1b8 (11, 16). *Ex vivo* studies revealed that both human AKR1B1 and -B10 also share the ability to reduce isocaproaldehyde (1, 40). Nevertheless, in a comparative enzymatic study, Hara and colleagues showed that AKR1B1 had a more effective isocaproaldehyde reductase activity than AKR1B10, suggesting that in human steroidogenic organs, the latter was unlikely to play a major role in the detoxification of steroidogenic by-products (41).

The AKR superfamily has been added as a novel group of cytosolic enzymes that could contribute to retinoid–redox conversion. Based on their cofactor specificity (NADPH), AKR work in the reductive direction (42). Retinol (vitamin A) and its derivatives, retinaldehyde and retinoic acid (RA), are essential for the growth and maintenance of many tissues. RA is a key molecule in the development of different vertebrate organs by promoting cell differentiation and apoptosis. The control of retinaldehyde levels is essential in the regulation of RA synthesis and therefore of its signaling role. Once synthesized from β-carotene through the β-carotene 15,15′ monooxygenase 1 (BCO1), retinaldehyde has two alternative fates, its irreversible oxidation to RA (metabolism fate) by the aldehyde dehydrogenases (ADH) or its reduction back to retinol (storage fate) by the retinaldehyde reductase activity of AKR (43) (Figure 3B; Table 4). Comparative *in vitro* enzymatic studies on murine and human AKRs have fairly evidenced that among AKR1B proteins, AKR1B10 is so far the only retinaldehyde reductase with the highest *k*_cat_ value for the retinaldehyde reduction (11, 12, 44). *Ex vivo* AKR1B10 overexpression in different cell systems demonstrated its contribution in increasing retinol production (45, 46). In rodent, previous data evidenced that normal adrenal gland may function as an important site of retinoic acid synthesis involving class I- and IV-ADH, thus furthering retinaldehyde metabolism rather than its storage through the AKR activity (47). According to its expression, whether the well-established retinaldehyde reductase activity of AKR1B10 is operated in normal human adrenal physiology remains to be explored.

*AKR1B10* expression was initially characterized in hepatocellular carcinoma and subsequently found to be altered by tumorigenesis process in several other organs (7, 48–50). Moreover, *AKR1B10* expression was associated with smoker’s non-small cell lung carcinomas (48) and was suggested to be involved in drug resistance (51). A putative mechanism by which the activity of AKR1B enzymes could promote tumor growth is the conversion of retinaldehyde to retinol resulting in RA deprivation and blockage of its differentiating effect, promoting cell proliferation and fostering tumorigenesis (43). Furthermore, recent studies have shown that in breast cancer cells, AKR1B10 associates with acetyl-CoA carboxylase-alpha (ACCA), the rate-limiting enzyme of *de novo* synthesis of long-chain fatty acids, and blocks its ubiquitination and proteasome degradation. Long-chain fatty acids are the building blocks of biomembranes and the precursor of lipid second messengers, playing a critical role in cell growth and proliferation (52). The AKR1B10-mediated regulation on ACCA stability represents a novel regulatory mechanism, in which AKR1B10 promotes cell survival *via* modulating lipid synthesis, mitochondrial function, oxidative stress, and carbonyl levels (53).

Adrenocortical carcinomas are very aggressive and rare malignant tumors with poor prognosis (54). Microarray analysis was used to seek molecular predictors of malignancy and survival in a large cohort of unilateral adrenocortical tumors (http://www.ebi.ac.uk/arrayexpress, experiment E-TABM-311). Unsupervised clustering analysis allowed robust discrimination of malignant and benign tumors. On the basis of this analysis, *AKR1B10* expression was not found to be associated with the ACC group (35).

### Akr1b7: Expression Profile, Detoxification Function, and Paracrine Action

High levels of *Akr1b7* transcripts were initially observed by *in situ* hybridization in fetal and adult murine adrenal cortex but were undetectable in the medulla (2). We confirmed these results by immunohistochemistry experiments, which allowed us to further restrict major Akr1b7 expression to the *zona fasciculata* (55). *In vivo*, ACTH suppression with dexamethasone treatment resulted in a marked decrease of *Akr1b7* mRNA levels that were restored when the treated mice were injected with exogenous ACTH. This ACTH/cAMP-induced *Akr1b7* transcription was blocked by a PKA inhibitor (H89) in the murine adrenocortical ATC and Y1 cell lines (55, 56).

In the adrenal gland, basal and ACTH-induced expressions of *Akr1b7* gene depend on three SF1 response element (SFRE) and on other *cis*-elements located in the upstream promoter region (Figure 3). Using transgenic mice and transfection experiments, we characterized a cryptic SFRE 102 bp upstream of the transcription start site that supports basal expression of *Akr1b7* in the adrenal cortex. Among the two other SFREs identified further upstream, the site at −458 was a *bona fide* SFRE, essential for both basal and cAMP-stimulated promoter activity. The last SFRE, an Sp1, and C/EBPβ binding sites, respectively, localized at positions −503, −52, and −61 are all involved in cAMP responsiveness (57, 58).

AKR1Bs are capable to handle the large amount of isocaproaldehyde, a toxic by-product coming from the cholesterol side-chain cleavage during the initial step of steroid biosynthesis. Furthermore, isocaproaldehyde accumulation decreased viability of Y1 cells (29). Although Akr1b3, Akr1b7, and Akr1b8 all were able to reduce isocaproaldehyde, the two former seemed to be the more efficient reductases for this substrate (28). However, the silencing of *Akr1b7* gene was sufficient to abolish the cAMP-induced isocaproaldehyde reductase activity in Y1 cells. Therefore, Akr1b7 was the main enzyme in charge of isocaproaldehyde detoxification in the adrenal cortex (29). Altogether, these data showed that in the adrenal cortex, ACTH not only controls expression of enzymes synthetizing steroids but also of proteins scavenging toxic by-products derived from steroidogenesis.

Madore et al. established that the bovine 20α-hydroxysteroid dehydrogenase, AKR1B5 was responsible for PGF_2α_ synthesis in the endometrium (59). Thereafter, we demonstrated by *ex vivo* studies, that AKR1B1, Akr1b3, and Akr1b7 were also able to reduce PGH_2_ into PGF_2α_, whereas Akr1b8 and AKR1B10 were devoid of this PGF_2α_ synthase activity (Table 4). Due to their recent discovery, this 9-,11-endoperoxide reductase activity has not been investigated yet for Akr1b16 and AKR1B15. Moreover, their enzymatic constants suggested that AKR1B1, Akr1b3, and Akr1b7 had a higher 9-,11-endoperoxide reductase activity than the other PGF synthases already described (34). Prostaglandins are paracrine/autocrine signal molecules produced from a common precursor, PGH_2_, which is derived from arachidonic acid by COX-1 or COX-2. Unlike COX-1, which is a constitutively expressed enzyme, COX-2 is not expressed in most organs under basal conditions but can be stimulated by inflammation and various mitogenic factors (60). Following these observations, we carefully examined the PGF_2α_ biosynthetic pathway in the adrenal gland (27).

Prostaglandin F2α was produced by both cortical (steroidogenic cells) and medullary (chromaffin cells) tissue of the adrenal gland. In primary adrenocortical cell culture, PGF_2α_ release was induced 2.5-fold by ACTH treatment. This secretion was correlated with ACTH responsiveness of both COX-2 and Akr1b7. Using *ex vivo* gain- and loss-of-function strategies, we demonstrated the pivotal role of Akr1b7 in ACTH-induced PGF_2α_ release, and it is functionally coupled with COX-2. In the adrenal medulla in which *Akr1b7* was not expressed, PGF_2α_ was produced from the coordinated activities of Akr1b3 and COX-1. Adrenal expression of PGF_2α_-specific receptor (FP) was restricted to the chromaffin cells, suggesting that both autocrine and paracrine mechanisms (within the medulla and between steroidogenic and medulla cells, respectively) were relaying PGF_2α_ action. In agreement with this hypothesis, we demonstrated that PGF_2α_ repressed both basal and glucocorticoid-induced dopamine release in the chromaffin cell line MPC862L. Comparison of the PGF_2α_-responsiveness of isolated cells and whole adrenal tissue cultures showed that PGF_2α_-mediated repression of glucocorticoid release is an indirect mechanism relying on a decrease in catecholamine secretion, which in turn decreased cortical steroidogenesis.

**Figure 4:**
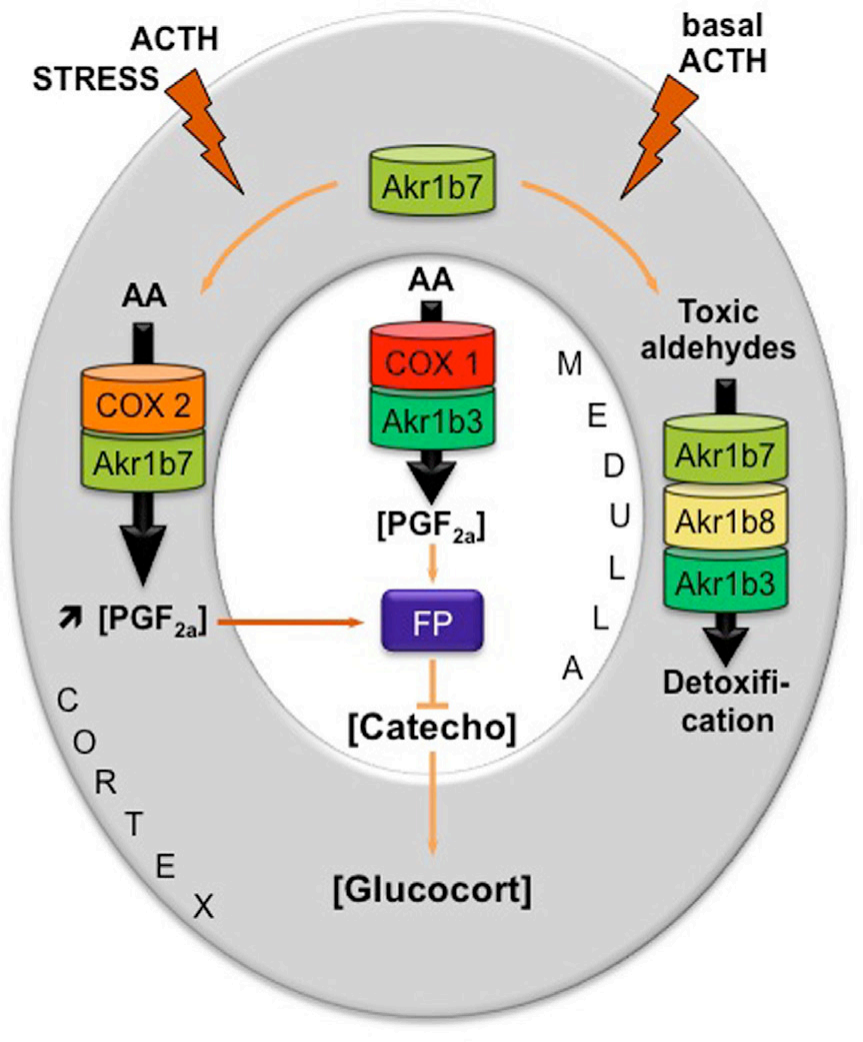
**Proposed model integrating dual functions of aldose reductases in the regulation of mouse adrenal endocrine functions [adapted from Ref. (18)]**. AKR1B family is endowed with enzymatic activities that at least ensure two metabolic functions: the production of PGF_2α_ and the detoxification of lipid aldehydes. Arachidonic acid (AA) is metabolized into PGH_2_, the precursor of all prostanoids, by COX enzymes and then converted into PGF_2α_ by the 9-,11-endoperoxyde reductase activity of PGF synthases of the AKR1B family. AKR1B are also capable to reduce toxic lipid aldehydes resulting from the particularly high prooxidant activities of P450 cytochromes in adrenocortical cells (i.e., isocaproaldehyde and 4-HNE). The mechanism integrating these dual functions was the following: (1) In basal conditions, PGF_2α_ is constitutively secreted by chromaffin cells (by the coupling of COX-1 and Akr1b3), thus regulating catecholamine production and also limiting their paracrine action on steroidogenesis. (2) During a stress situation, ACTH transiently induces COX-2 and Akr1b7 expression, which results in PGF_2α_ production inside the cortex. PGF_2α_ produced in the cortex then represses catecholamine release by the medulla *via* a paracrine action on its FP receptor. Decreased catecholamine release in turn reduces the effect of ACTH on glucocorticoids production (27). After the stress response has ended, COX-2 returns to undetectable levels. The coupling between Akr1b7 and COX-2 does not take place. Then, Akr1b7 together with Akr1b8 and Akr1b3 function only as cortical detoxifying enzymes of the harmful aldehydes produced under chronic/basal stimulation of steroidogenesis. Catecho, catecholamine; Glucocort, glucocorticoids.

These functional data led us to propose an intra-adrenal feedback loop in which adrenal endocrine activities are regulated through the involvement of AKRs [Figure 4 and Ref. (18)]. Surprisingly, however, the absence of Akr1b7 *in vivo* did not affect basal adrenocortical function as illustrated by normal glucocorticoid plasma levels in *Akr1b7*^−^*^/^*^−^ mice (61). Indeed, these mice displayed an obese phenotype that did not rely on adrenal dysfunction but on the lack of Akr1b7-dependent production of PGF_2α_ within the stromal vascular adipose tissue (3, 61). Adrenal expression of Akr1b3 and b8 is not affected in knockout mice and since they all share redundant enzymatic activities regarding detoxification of lipid aldehydes (see Akr1b3/AKR1B1: Expression Pattern and Relevant Functions and Akr1b8/AKR1B10: Phylogeny, Regulation, and Enzymatic Specificities), the remaining isoforms can compensate the absence of Akr1b7 at least in basal conditions. Importantly, Akr1b7 is the only one out of the three adrenal isoforms to be ACTH-responsive (27) and also the most abundantly expressed (3). Taken together, these hallmarks would predict that physiological importance of Akr1b enzymes in adrenal function should be rather explored under stress conditions during which scavenging capacity of constitutive (and less abundant) isoforms should be exhausted.

## Future Directions

Fighting against oxidative stress is a challenging but mandatory task for adrenocortical cells. Indeed, P450 cytochrome systems involved in steroidogenesis, and in particular glucocorticoid production, contribute very significantly to oxidative stress by cellular reactive oxygen species (ROS) production (62). The redox imbalance due to excessive ROS production can cause adrenal damage that may progress to severe insufficiency, including familial glucocorticoid deficiency (FGD). Therefore, adrenal cortex is well supplied in antioxidant defense genes encoding enzymes of the superoxide dismutase (SOD), glutathione peroxidase (GPX), and peroxiredoxin (PRDX) families [for review, see Ref. (63)]. Since the precursor works of Feige’s group showing the ACTH responsiveness of *SOD2* expression, the expected coordinated regulation of antioxidant enzymatic systems and P450s systems producing prooxidant by-products has been somewhat neglected (64). Accordingly, AKR1B enzymes family may be considered as antioxidant defense genes. Among these, ACTH-responsive ones, e.g., *Akr1b7* and *SOD2* genes, could participate in the adaptive response of antioxidant systems of adrenal cortex under stress conditions. Disturbance in redox homeostasis was the most recently discovered cause of FGD and mutations in *NNT* gene (nicotinamide nucleotide transhydrogenase) account for about 10% of cases (65). NNT ensures mitochondrial NADPH supply that is essential to ROS detoxification enzymatic systems. Then, it would be interesting to know whether adrenal-specific deficit in AKR1B enzymes could contribute to cortical damage or adrenal insufficiency in mice carrying a spontaneous *Nnt* mutation (66). This could provide the proof of principle for studying the physiological contribution of AKR1B family in detoxifying function in steroidogenic organs and beyond.

## Author Contributions

All authors listed have made substantial, direct, and intellectual contribution to the work and approved it for publication.

## Conflict of Interest Statement

The authors declare that the research was conducted in the absence of any commercial or financial relationships that could be construed as a potential conflict of interest.
